# Targeting DNA Damage Response in the Radio(Chemo)therapy of Non-Small Cell Lung Cancer

**DOI:** 10.3390/ijms17060839

**Published:** 2016-05-31

**Authors:** Ling Li, Tao Zhu, Yuan-Feng Gao, Wei Zheng, Chen-Jing Wang, Ling Xiao, Ma-Sha Huang, Ji-Ye Yin, Hong-Hao Zhou, Zhao-Qian Liu

**Affiliations:** 1 Department of Clinical Pharmacology, Xiangya Hospital, Central South University, Changsha 410008, China; yx132liling@126.com (L.L.); euzhutao@hotmail.com (T.Z.); gaoyuanfeng126@126.com (Y.-F.G.); zhengweiinxy@126.com (W.Z.); wangchenjing1117@126.com (C.-J.W.); xiaolingcsu@csu.edu.cn (L.X.); martha0126@163.com (M.-S.H.); yinjiye2005@sina.com (J.-Y.Y.); hhzhou2003@163.com (H.-H.Z.); 2 Institute of Clinical Pharmacology, Hunan Key Laboratory of Pharmacogenetics, Central South University, Changsha 410078, China

**Keywords:** non-small cell lung cancer, DNA damage and repair, radiotherapy and chemotherapy, resistance, genetic polymorphisms, epigenetics

## Abstract

Lung cancer is the leading cause of cancer death worldwide due to its high incidence and mortality. As the most common lung cancer, non-small cell lung cancer (NSCLC) is a terrible threat to human health. Despite improvements in diagnosis and combined treatments including surgical resection, radiotherapy and chemotherapy, the overall survival for NSCLC patients still remains poor. DNA damage is considered to be the primary cause of lung cancer development and is normally recognized and repaired by the intrinsic DNA damage response machinery. The role of DNA repair pathways in radio(chemo)therapy-resistant cancers has become an area of significant interest in the clinical setting. Meanwhile, some studies have proved that genetic and epigenetic factors can alter the DNA damage response and repair, which results in changes of the radiation and chemotherapy curative effect in NSCLC. In this review, we focus on the effect of genetic polymorphisms and epigenetic factors such as miRNA regulation and lncRNA regulation participating in DNA damage repair in response to radio(chemo)therapy in NSCLC. These may provide novel information on the radio(chemo)therapy of NSCLC based on the individual DNA damage response.

## 1. Introduction

Non-small cell lung cancer (NSCLC) accounts for about 85% of lung cancer cases. NSCLC is the leading cause of cancer death worldwide due to its high incidence and mortality for both men and women, and it has become a serious health problem [1]. It is widely accepted that lung cancer development is the result of many factors, such as genetic, environmental, food and lifestyle factors (particularly smoking related), among which DNA damage is considered to be the primary cause of lung cancer [2]. The systemic treatments for lung cancer consist of classical surgery, standard radiotherapy and chemotherapy, and monotherapy, and a combination therapy of these approaches is recommended in clinical practice. One main impediment for treating NSCLC is that most patients are diagnosed at an advanced stage, consequently missing the most opportune window for surgical intervention. So radiotherapy and chemotherapy become important therapeutic approaches for unresectable NSCLC [3]. Radiotherapy and platinum-based chemotherapy play key roles in the treatment of NSCLC by damaging DNA and inducing tumor cell death. However, regardless of these conventional therapies, the five-year survival rate of NSCLC has still not improved significantly, remaining at less than 15% [4]. In addition, clinical resistance (intrinsic or acquired) to radio(chemo)therapy is considered as another impediment in the treatment of human NSCLC, which can be attributed to many factors. One such factor is the DNA repair capacity of damaged cells in mediating resistance to radiation and platinum-based chemotherapy [5,6]. Thus, the roles of DNA repair pathways in radio(chemo)therapy-resistant cancers have attracted widespread interest in clinic.

**Figure 1:**
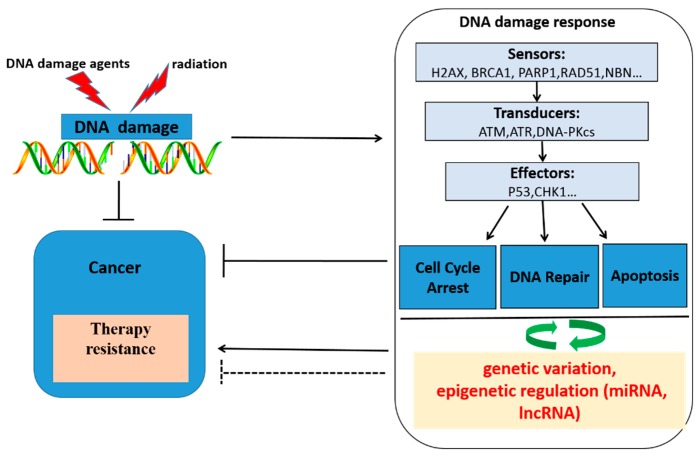
Schematic overview of DNA damaging agents, radiotherapy, DNA damage, the cellular response upon DNA damage, genetic variation, epigenetic regulation and cancer therapy response. Genotoxic agents and radiation induce DNA damage, which activates the DNA damage response. The DNA damage response consists of three steps processes: At the first time, DNA damage was recognized by DNA damage association proteins, then transducing damage signals to the cell, lastly, cell cycle arrest, DNA repair and apoptosis biological process were activated by DNA damage. Thus, cancer cells will be killed. DNA damage response became a therapeutic target. However, tumors can develop therapy resistance by activating or inhibiting various processes within the DNA damage response by a bidirectional regulatory pathway exists between DNA damage response and miRNAs, lncRNA, genetic variation in DNA repair genes, which will influence DNA damage response, then regulation radio(chemo)therapy response.

An increasing number of studies have shown that large inter-individual variability exists in the capacity of DNA damage response and DNA repair, and sensitivity to radiation and chemotherapy varies from person to person, thus generating different therapeutic effects despite uniform treatment protocols [7,8]. Some studies have shown that genetic factors play a leading role in the inter-individual variation in radio(chemo)therapy sensitivity [9,10,11]. The expression level of DNA repair genes and the activity of the encoded proteins determine whether or not radio/chemo-resistance will occur by determining the capacity of DNA repair. In NSCLC, a deficiency in DNA repair could be a result of a mutation in one DNA repair gene, but much more frequently reduced DNA repair genes or the absent expression of DNA repair genes could be attributed to epigenetic alterations that reduce or silence corresponding gene expression. With the evolution of DNA sequencing and bioinformatics, genetic variations and epigenetic modification, such as miRNA and lncRNA regulation, have emerged as a novel research field in DNA repair pathways which influence radio(chemo)therapeutic outcome. In order to gain insights into the underlying mechanism of radio(chemo)therapy resistance in NSCLC, this review focuses on the various DNA repair genes from two aspects: genetic and epigenetic levels, with a particular emphasis on their role in mediating radio(chemo)therapy resistance in NSCLC. Understanding the DNA repair mechanism has huge implications for potentiating the anti-tumor effect of radiotherapy and genotoxic chemotherapy, helping to achieve personalized therapy for NSCLC (Figure 1).

## 2. Initiation of the DNA Damage Response and DNA Repair Pathway

DNA is vulnerable to damage that originates from endogenous metabolites, such as macrophages and neutrophils produced ROS (reactive oxygen species), RNS (reactive nitrogen species) [12] and exogenous agents including smoking, chemical carcinogens, radiation [13], and genotoxic cancer therapeutics [14]. For instance, platinum compounds such as cisplatin can form the platinum-DNA (Pt-DNA) adducts in the DNA which introduces covalent links between bases of the same or different DNA strands (intra-strand crosslinks or inter-strand crosslinks) [15]. Additionally, radiation may induce single-strand or double-strand DNA breaks (SSBs and DSBs) by inducing the oxidation of DNA bases causing lesions in the DNA. These lesions give rise to gene mutations and chromosomal damage, which are causal events in oncogenic transformation, malignant progression and radio(chemo)therapy resistance [16,17]. DSBs have the most harmful effect on cell survival among all DNA lesions, and are strong activators of apoptosis [18]; cell death could be induced by the persistence of DSBs if not repaired. To preserve genomic stability and survival, cells have developed DNA damage response (DDR) to handle DNA lesions [19]. When the genome gets damaged by radiation or chemotherapy, cells have to initiate an efficient response to faithfully repair the lesion and maintain genome integrity [20].

DNA repair is orchestrated by a series of pathways, mainly including nucleotide excision repair (NER), base excision repair (BER), DNA mismatch repair (MMR) and single-strand break repair (SSBs) [21]. Among these DNA repair pathways, NER repairs damaged DNA commonly caused by chemotherapeutics such as platinum drugs, which has been proven to be associated with chemotherapy resistance in NSCLC [22]. The repair of DSBs is carried out by two major repair pathways: non-homologous DNA end joining (NHEJ) and homologous recombination (HR). NHEJ acts on the G0/G1 cell cycle phase, and HR plays a role in the late S/G2 phase [19]. Blocking these repair pathways will likely result in increased radiotherapy and chemotherapy sensitivity.

In order to ensure that cells respond to DSBs quickly and accurately, three steps are supposed to be necessary: Firstly, the damage must be detected as soon as possible. Three interconnected sensor systems have been described that have the ability to detect a single DSB within minutes after its formation [23]. These sensors in the DDR are the PI3K-related kinases (PIKKs): ataxia telangiectasia and Rad3-related (ATR), ataxia telangiectasia mutated (ATM) and DNA-dependent protein kinase (DNA-PK). ATR plays a key role in recognition of SSBs induced by cisplatin or IR, while ATM is mainly involved in the recognition of DSBs. Secondly, damage signals are transduced to the cell. Lastly, cells react to decide to either repair damaged DNA or to activate cell cycle checkpoints or induce apoptosis. Once DSBs are induced by cisplatin or IR, as a transducer, ATM/ATR targets many dual-function proteins that are key nodes in promoting survival or cell death. Then these proteins signal downstream checkpoint activation to prevent cell cycle progression that provides time for the cell to repair the damage and the recruitment of DNA repair proteins to facilitate DSB repair via the stimulation of NHEJ or HR, depending on the cell cycle phase [24]. When the damage to DNA is greater than the repair capacity, the remaining DNA damage will block replication and transcription, and activate DDR signals downstream cell death pathways. The repair capacity of the cell, the status of p53 and key DDR proteins including ATR, ATM and DNA-PK, the effectiveness of activating DNA repair genes, and the execution of downstream cell death pathways are determinants of a cell surviving death. The expression levels of these genes including *XPA*, *ERCC3/XPB*, *XPC*, *ERCC2/XPD*, *DDB1/XPE*, *ERCC4/XPF*, *ERCC5/XPG*, *ERCC1*, *XRCC* family, *ATM*, *NBN*, *BRCA1*, *RAD51*, *P53*, and *EIF3A* are important for the repair capacity and the response to radiotherapy and chemotherapy [25,26]. Although mutations of DNA repair genes and epigenetic alterations involved in DNA repair process are occasionally occurring events in cancer, they will change the expression of DNA repair genes, alter DNA repair capacity, and hence affect the response to radio(chemo)therapy in NSCLC.

## 3. Association of Genetic Polymorphisms in DNA Repair Genes with Radio(chemo)therapy Response in Non-Small Cell Lung Cancer (NSCLC)

Genetic variations in DNA repair genes are thought to modulate DNA repair capacity and be related to the sensitivity of radio(chemo)therapy in NSCLC. Thus, certain single nucleotide polymorphisms (SNPs) may be used to predict the sensitivity or clinical outcomes of radio(chemo)therapy in patients, which may play an important role in the individualized treatment of NSCLC. Through a number of case-control studies, SNPs at candidate loci were genotyped across patients with or without radio(chemo)therapy resistance and toxicity, and many genetic markers have been investigated in human NSCLC [27,28,29,30,31]. In the following text, we briefly recapitulate our knowledge of these SNPs in DNA repair genes, and how they play roles in radio(chemo)therapy response (Table 1).

### 3.1. X-ray Cross-Complementing (XRCC) Family Genes

The *XRCC* (X-ray cross-complementing) genes are discovered mainly through their roles in protecting mammalian cells from damage caused by ionizing radiations and anti-tumor chemotherapy agents. In the *XRCC* family, *XRCC1*, *XRCC2*, *XRCC3*, *XRCC4* and *XRCC5* are studied frequently in cancers. Among these genes, *XRCC1* is the most frequently researched gene. It is a limiting factor in the BER pathway. *XRCC1* is over-expressed in NSCLC [32], and the expression levels of *XRCC1* have shown a significant correlation with cisplatin chemo-resistance in NSCLC cell lines [33]. Changes in the amino acids will affect the normal function of XRCC1 protein and cause altered DNA repair activity [34,35,36,37]. The Arg194Trp and Arg399Gln genetic polymorphisms are the most extensively studied SNPs of the *XRCC1* gene. Evidence from lung cancer patients showed that *XRCC1* (rs25487, Arg399Gln) might influence radiation and platinum-based chemotherapy wherein patients with the ancestral allele (G) were found to be more radiosensitive and have a higher overall survival [38], while the *XRCC1* 399Arg/Arg genotype carriers had a higher response rate than that of the Gln genotype carriers (OR = 4.81, 95% CI = 1.778–13.013, *p* = 0.002) [39,40] and a poor overall survival in a short-term period (HR = 1.718, *p*  =  0.003; HR = 1.691, *p*  =  0.003, respectively) in a treatment with platinum-based chemotherapy for NSCLC patients [41]. As for the *XRCC1* Arg194Trp polymorphism, it was found to be significantly associated with better response rates to platinum-based chemotherapy in advanced NSCLC [36,42,43].

*XRCC2* and *XRCC3* genes encode a member of the RecA/Rad51-related protein family that is involved in the repair of DSBs by the HR pathway [44]. Previous findings demonstrated that *XRCC2* (rs3218536, Arg188His) was correlated with overall survival (OS) in NSCLC patients treated with radiotherapy [45]. A meta-analysis result showed that the *XRCC3* Thr241Met polymorphism had an impact on the response to platinum-based chemotherapy in patients with advanced NSCLC and *XRCC3* carriers of the variant 241Met allele were significantly associated with better response [46]. In addition, one study showed that male patients with the TT genotype of *XRCC4* rs6869366 (−1394G>T) and female patients with the AG/AA genotypes of *XRCC5* rs3835 (2408G>A) were at increased risk of severe radiation-induced pneumonitis in NSCLC [47]. The use of *XRCC* family genes’ polymorphisms as predictors of clinical outcomes in personalized radio(chemo)therapy treatment requires further verification from large, well-designed pharmacogenetics studies.

### 3.2. Excision Repair Cross-Complementing (ERCC) Family Genes

*ERCC* (excision repair cross-complementing group) family genes mainly include *ERCC1*, *ERCC2* (also named *XPD*), *ERCC3* (also named *XPB*), *ERCC4* (also named *XPF*) and *ERCC5* (also named *XPG*), and they participate in DNA repair and DNA recombination. *ERCC1* expression has proved to be related to the clinical benefit of platinum-based chemotherapy [6,48,49]. High *ERCC1* expression is associated with a significantly worse OS in platinum-treated NSCLC patients [50]. Genetic polymorphisms of *ERCC1* may alter the repair function, possibly by changing its expression level. Previous studies have demonstrated that the G/G genotype of *ERCC1* rs11615 is associated with a better survival [51] and higher sensitivity to cisplatin in advanced NSCLC patients [52].

In addition, *ERCC2* rs13181 has been studied regarding its effect on platinum-based chemotherapy sensitivity. A meta-analysis of clinical studies has shown that *ERCC2* rs13181 with A/C and C/C genotypes is associated with low sensitivity in Asian populations and with high sensitivity in Caucasian NSCLC patients that were treated with platinum drugs [53]. Thus, *ERCC2* Lys751Gln (A>C) may act as a predictor in NSCLC treated with platinum-based chemotherapy according to different ethnicities. More recently, another study has reported that patients with the *ERCC3* rs3738948 G/G or G/A genotype show a better response, and those with the *ERCC5* rs2296147 TT genotype and the T allele show a significantly reduced risk of developing progressive NSCLC when receiving platinum-based chemotherapy, while advanced NSCLC patients carrying the *ERCC5* rs2094258 G/G and the G allele show a significantly decreased risk of developing a progressive disease [54,59]. There is little evidence that SNPs in other *ERCC* family genes have potentials to be used as predictors of radiotherapy or chemotherapy and more research is needed.

### 3.3. Ataxia Telangiectasia Mutated (ATM)

ATM (Ataxia telangiectasia mutated) is a serine/threonine protein kinase, a key DDR kinase and tumor suppressor, that is recruited and activated by DSBs induced by ionizing radiation or genotoxicity drugs [60]. It plays a central role in the recognition and signaling of DNA damage by regulating several key proteins (such as p53, CHK1, BRCA1, NBN, H2AX, ARF) that initiate activation of the DNA damage checkpoint, leading to cell cycle arrest, DNA repair or apoptosis [60,61]. Recently, a functional interplay between ATM and the alternative reading frame (ARF) tumor suppressor protein in response to oncogenic insults has been shown. ATM can suppress ARF protein levels and activity in a transcription-independent manner [62,63]. Loss of ATM will render tumors resistant to the cell death pathway.

Accompanied by a gain of therapeutic resistance, *ATM* is frequently mutated in tumors [64]. Some independent studies have shown the ATM (rs189037 (G>A)) A allele as a risk allele for radiation pneumonitis in NSCLC patients upon radiotherapy [55,56,57]. Additionally, further studies demonstrated that rs189037 might affect ATM expression by reducing transcriptional activity and interfering nuclear protein binding *in vitro* and *in vivo* [56]. In addition, haplotype analysis showed that patients carrying *ATM* rs228590 TT/CT or rs189037 AG/GG genotypes or rs228590T/rs189037G/rs1801516G (G-T-G) haplotypes had a lower risk of severe radiation pneumonitis when receiving definitive radio(chemo)therapy [57,58].

## 4. Noncoding RNAs and Radio(chemo)therapy Response in NSCLC

**Figure 2:**
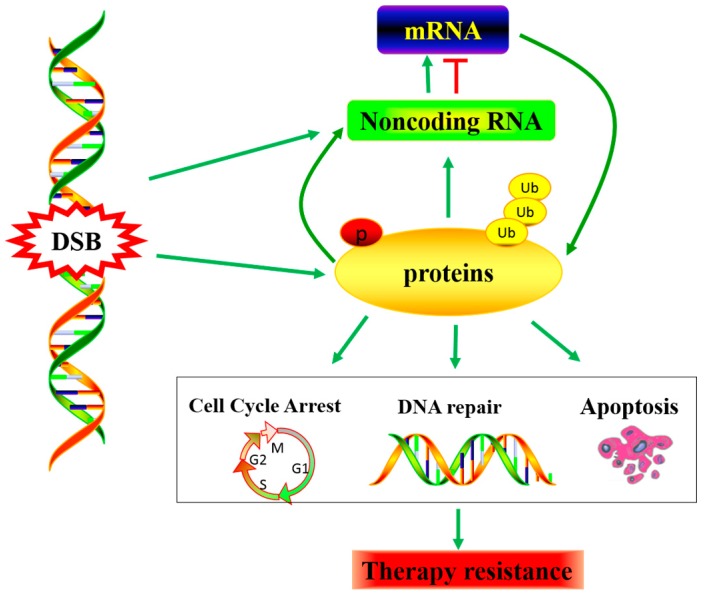
DNA damage-induced non-coding RNA dys-regulating, Noncoding RNA as a connectivity node between the rapid DNA damage response mediated by protein modifications and the late response mediated by transcriptional regulation. Consequently, affecting the radiation and chemotherapy effect.

In the past, the most common approach to identifying genetic markers of drug response focused on SNPs [65]. However, along with the rapid development of sequencing technology and bioinformatics, epigenetic alterations have been increasingly identified and recognized as a frequent event in cancers. Non-coding RNAs (ncRNAs) are now widely thought to be critical in the biological processes of cancer incidence and radio(chemo)therapy sensitivity. Additionally, as a new epigenetic regulation, it affects the sensitivity of radio(chemo)therapy by regulating the DDR pathway through modulating the expression or location of key genes, such as cell cycle control genes, DNA repair genes and apoptosis genes. Thus, ncRNAs have great potential to be used as biomarkers for personalized therapy in NSCLC. Next, we will put emphasis on microRNAs and lncRNAs, summarize how they play roles in DDR to alter sensitivity to radio(chemo)therapy in NSCLC, and discuss the potential clinical applications of ncRNAs as predictors or therapeutic targets for NSCLC (Figure 2).

### 4.1. MicroRNAs and Radio(chemo)therapy Response in NSCLC

MicroRNAs (miRNAs) are a family of small non-coding RNAs that range in size from 19 to 25 nucleotides and negatively regulate the expression of their target genes at the post-transcriptional level, leading to a translational repression or mRNA degradation [66]. MicroRNAs are involved in radio(chemo)therapy resistance through DNA repair mechanisms [67,68]. Additionally, mRNAs are required for almost every aspect of cellular responses to DNA damage, including sensing DNA damage, transducing damage signals, repairing damaged DNA, activating cell cycle checkpoints, and inducing apoptosis [68].

With the development of gene chip technology and high throughput technology, gene expression profiles have been widely used to detect differentially expressed genes in DNA damage response. Accumulating evidence suggests that altered expression levels of candidate miRNAs may be involved in the acquisition of tumor cell resistance to radiotherapy and conventional chemotherapy through impacting the efficacy of DNA repair. For example, one study showed that 14 miRNAs were found dysregulated in doxorubicin-resistant A549 cells compared to A549 cells. Of these 14 miRNAs, four (has-mir-1973, 494, 4286 and 29b-3p) showed a 2.99- to 4.44-fold increase in their expression. Similarly, alteration of miRNA profiles was detected in A549 cells upon ionizing radiation [69]. These data suggest that miRNA expression is affected by DNA damage, and they may be involved in the regulation of DNA damage repair. This holds implications for using miRNA expression as resistance markers/targets to improve responses to chemotherapeutic drugs and radiotherapy [70].

DDR regulates miRNA expression via multiple mechanisms. Interestingly, recent studies have shown that a bidirectional regulatory pathway exists between miRNAs and DDR. In response to DNA damage, miRNAs can directly regulate cellular processes involved in DDR by altering their target genes, which provides a feedback regulatory loop for miRNA-mediated DDR [71]. For instance, the tumor suppressor p53 is a key factor that is induced by DNA damage. Various miRNAs are involved in the p53 network [72]. Among these miRNAs, the miR-34 family directly and post-transcriptionally targets p53, and they are activated by ionizing radiation (IR) or capsaicin-induced oxidative DNA damage [73,74]. Further functional analyses showed that the p53/miR-34a regulatory axis might be critical in sensitizing drug-resistant NSCLC cells [74]. MiR-34a can directly bind to the 3′ untranslated region of RAD51 and regulates homologous recombination, and inhibits DSB repair in NSCLC cells after DNA damage [75]. A recent study showed that miR-15b/16-2 can promote p53 phosphorylation and promote the DNA damage response through inhibiting Wip1 (PPM1D) expression following radiation in lung epithelial cells. Additionally, in miR-15b/16-2-overexpressing cells the ATM/Chek1/p53 pathway was activated by IR [76].

At the same time, some miRNAs can regulate the formation of cancer stem cells and the acquisition of the epithelial-mesenchymal transition (EMT), which is critically associated with drug resistance [77]. For instance, a recent study reported that miR-138 could reduce the DNA damage repair capacity of small cell lung cancer (SCLC) cells by targeting *H2AX* which plays an important role in DNA repair by increasing the local concentration of repair factors near the lesion [78]. Another typical example is *CDC6*, which is frequently overexpressed in lung cancer and leads to genomic instability and EMT [79]. *CDC6* over-expression can give rise to a shortened 3′ UTR which lacks miRNA binding sites, thus increasing its mRNA stability [80]. Moreover, some miRNAs could target drug sensitivity-related genes. For example, miR-630 emerged as a novel modulator of the DNA damage response to cisplatin by targeting ATM kinase and the ATM substrates histone H2AX and p53. Therefore, it induces cell cycle arrest, counteracts early events of the response to DNA damage and results in the greatly diminished sensitivity of cisplatin in A549 NSCLC cells [81]. In addition, miR-138 plays a role in causing resistance to cisplatin in NSCLC by negatively regulating the *ERCC1* gene which is involved in the NER pathway [82]. Furthermore, miR-513a-3p can sensitize A549 NSCLC cells to cisplatin by targeting GSTP1 (Glutathione *S*-Transferase P1), which has been reported to contribute to cisplatin resistance in many studies [83]. These results suggest that miRNAs play important roles in DDR, which may affect the sensitivity to chemotherapy or radiotherapy. Therefore, miRNA-based therapeutics provide an attractive anti-tumor approach for developing new and more effective individualized therapeutic strategies and for predicting the response to different anti-cancer drugs which target DDR and DNA repair. The differentially expressed miRNAs in DDR may become a novel strategy to overcome chemo(radio)therapy resistance for the treatment of NSCLC.

### 4.2. Long Non-Coding RNAs (LncRNAs) and Radio(chemo)therapy Response in NSCLC

Long non-coding RNAs (lncRNAs) are simply defined as ncRNAs of length greater than 200 nucleotides that are not translated into proteins [84]. However, a further large-scale sequencing study provides evidence that many transcripts thought to be lncRNAs may, in fact, be translated into proteins [85]. Although tremendous advances have been made to elucidate the role of lncRNAs, their functions are largely unknown and difficult to determine. For instance, their effect on genome integrity maintenance is not well understood. LncRNAs can interact with DNA, RNA, and proteins. Recently studies have shown that lncRNAs control the transcription of genes relevant to DNA damage response by four different regulatory models, including signal, decoy, guide, and scaffold [86,87]. It is well known that DNA damage response and repair ability is closely related to the sensitivity to radio(chemo)therapy. Thus, lncRNAs that regulate DDR may become biomarkers for DNA-damaging anti-cancer treatments including radiation and chemotherapy in NSCLC.

In the effort to understand the contribution of lncRNAs to the DNA damage repair pathway, microarray becomes a useful tool. For instance, in order to identify those non-coding transcripts expressed in a p53-dependent manner, Huarte *et al.* [88] designed an experiment using gene chip technology to prove the contribution of lncRNAs in a mouse model in which p53 was activated by DNA damage. They found that lincRNA-p21 (Trp53cor1) was involved in DNA damage and cell cycle control. LincRNA-p21 serves as a repressor in p53-dependent transcriptional responses, on the contrary, and inhibiting lincRNA-p21 affects the expression of hundreds of gene targets which are normally repressed by p53 [88], and lincRNA-p21 is required to trigger p53-dependent apoptosis following DNA damage by binding to heterogeneous nuclear ribonucleoprotein K (hnRNP-K); then lincRNA p21 is recruited to the promoters of genes and represses their expression in a p53-dependent manner [89]. LincRNA-p21 knockout mice present decreased expression of cdkn1a in cis-regulation to promote target gene expression and to enforce the G1/S checkpoint causing increased proliferation [90]. In addition, Sharma *et al.* [91] identify that expression of the DNA damage-induced lncRNA (DDSR1) is induced in an ATM-NF-κB pathway—dependent manner by several DNA double-strand break (DSB)-inducing agents. Additionally, DDSR1 can increase DNA repair capacity by homologous recombination (HR) through interacting with BRCA1 and hnRNPUL1 [91]. Thus, these studies suggest that lncRNAs play critical roles in the DDR pathway by regulating gene expression or modulating repair capacity.

Recently, numerous lncRNAs have been shown to correlate with drug resistance in lung cancer in a p53-dependent or independent manner. For instance, Lnc_bc060912 were proved to be regulated by p53 [92], and to suppress cell apoptosis via interacting with the two DNA damage repair proteins PARP1 and NPM1 [92], while MEG3 can regulate cisplatin resistance through controlling the expression of p53 and Bcl-xl in lung adenocarcinoma cells [93]. In addition, the long non-coding RNA HOTAIR contributes to cisplatin resistance in human lung adenocarcinoma cells via interaction with EZH2 which leads to chromosome modifications and the down-regulation of p21 (WAF1/CIP1) expression [94]. In addition, cisplatin resistance in NSCLC has also been associated with other lncRNAs such as AK126698 [95]. Additionally, more lncRNAs involved in DNA damage repair and drug resistance or radio-resistance of lung cancer cells are likely to be discovered in the further studies. Thus, lncRNAs may become biomarkers for improving the sensitivity and specificity of lung cancer treatment in the near future.

## 5. Conclusions and Perspectives

DNA repair is a vital target to improve NSCLC therapy and to reduce the resistance of tumor cells to chemotherapy or radiotherapy. We have discussed the factors affecting the DNA repair capacity from genetic and epigenetic aspects including single nucleotide polymorphisms and miRNA and lncRNA regulation in the DNA repair pathway. The involvement of these factors in DNA damage repair—induced chemo-resistance is an emerging area and many key questions need to be investigated further: (1) Many positive results have been reported in genetic polymorphism studies but they are always inconsistent. Thus, evidence from large-scale clinical trials is needed, and further biological mechanism research is necessary to understand how and to what degree a genotype will affect a phenotype; (2) Current research regarding the roles of miRNA and lncRNA regulation in the DNA repair pathway is still limited, and thus further study to test their contribution to DNA damage repair is highly needed. Regulation of the DNA repair pathway by ncRNAs offers opportunities to develop potential predictors for radio (chemo) sensitivity and therapeutic targets for NSCLC.

## Figures and Tables

**Table 1:** Evidence for correlation of genetic variants with radio(chemo)therapy response in Non-Small Cell Lung Cancer (NSCLC).

Host Gene	SNP Site	Therapy Method	Effect	Reference
*XRCC1*	rs25487 (G399A)	Radiation	Patients with the ancestral allele (G) were found to be more radiosensitive	[38]
*XRCC1*	rs25487 (G399A)	Platinum-based chemotherapy	AA genotype patients presented higher response rates and had higher risk of hematologic toxicity toward platinum drug treatment compared with G model	[39,40,41]
*XRCC1*	rs1799782 (Arg194Trp)	Platinum-based chemotherapy	Patients with the TrpTrp and TrpArg genotypes were more likely to have better response rates to platinum-based chemotherapy	[42,43,44]
*XRCC2*	rs3218536 (Arg188His)	Radiation	Correlated with overall survival (OS) in NSCLC patients treated with radiotherapy	[45]
*XRCC3*	rs861539 (Thr241Met)	Platinum-based chemotherapy	*XRCC3* carriers of the variant 241Met allele were significantly associated with better response	[46]
*XRCC4*	rs6869366 (G1394T)	Radiation	G allele of *XRCC4* showed a tendency towards a decreasing risk of severe radiation pneumonia	[47]
*XRCC5*	rs3835 (G2408A)	Radiation	*XRCC5* rs3835 SNP showed significantly higher risk of developing severe RP	[47]
*ERCC1*	rs11615 (C>T)	Platinum-based chemotherapy	T/T genotype associated with low sensitivity, GG genotype was associated with a better survival	[51,52]
*ERCC2/XPD*	rs13181 (G>T)	Platinum-based chemotherapy	*ERCC2* rs13181 with C allele associated with low sensitivity in Asian populations and high sensitivity in Caucasian NSCLC patients that were treated with platinum drugs	[53]
*ERCC3*	rs3738948 (A>G)	Platinum-based chemotherapy	Patients with G allele achieved better response	[54]
*ERCC5*	rs2296147 (C>T); rs2094258 (A>G)	Platinum-based chemotherapy	Patients with rs2296147 T allele and rs2094258 G allele had a significantly reduced risk of developing progressive NSCLC	[54]
*ATM*	rs189037 (G>A)	Radiation	A allele as a risk allele for radiation pneumonitis in NSCLC patients	[55,56,57]
*ATM*	rs228590 (C>T)	Radiation	Patients carrying T allele had a lower risk of severe radiation pneumonitis in NSCLC patients	[56,58]

## Abbreviations

The following abbreviations are used in this manuscript:

DSB	Double-Strand DNA Break
DDR	DNA Damage Response
NER	Nucleotide Excision Repair
NHEJ	Non-Homologous DNA End Joining
HR	Homologous Recombination
ATR	Ataxia Telangiectasia and Rad3-Related ATR Serine/Threonine Kinase
ATM	Ataxia Telangiectasia Mutated
DNA-PK	DNA-Dependent Protein Kinase
LncRNA	Long Non-Coding RNA
miRNA	MicroRNA
mRNA	Messenger RNA
EIF3A	Eukaryotic Translation Initiation Factor 3 Subunit A
RAD51	RAD51 Recombinase
CHK1	Checkpoint Kinase 1
H2AX	Histone H2A
PARP1	Poly [ADP-Ribose] Polymerase 1
NPM1	Nucleophosmin (Nucleolar Phosphoprotein B23, Numatrin)
EZH2	Enhancer of Zeste 2 Polycomb Repressive Complex 2 Subunit
BRCA1	Breast Cancer 1
NBN	Nibrin
SNPs	Single Nucleotide Polymorphisms
